# A non-randomised trial of video and written educational adjuncts in undergraduate ophthalmology

**DOI:** 10.1186/s12909-019-1923-1

**Published:** 2020-01-09

**Authors:** H. D. Jeffry Hogg, Michael Pereira, Julian Purdy, Richard J. R. Frearson, Gordon B. Lau

**Affiliations:** 1 The University of Newcastle upon Tyne, Newcastle upon Tyne, Tyne and Wear NE1 7RU UK; 2 The Newcastle upon Tyne Hospitals NHS Foundation Trust, Newcastle upon Tyne, Tyne and Wear NE1 7RU UK

**Keywords:** Undergraduate education, Ophthalmology, Learning resources, Technology enhanced learning, Video resources

## Abstract

**Background:**

Provision of relevant pre-learning materials has been shown to increase student engagement and improve outcomes in medical education. This non-randomised study attempts to quantify the educational gains, and relative efficacy of video and written pre-learning materials, in ophthalmology undergraduate teaching.

**Methods:**

Ninety-eight final year medical students were contacted prior to their three-day ophthalmology placements at a British tertiary ophthalmology unit. All participants were sent welcome packs prior to arrival requesting they undertake 90 min of work focusing on a list of specified ophthalmic conditions. One cohort (*N* = 33) were sent written materials, another (*N* = 32) was provided with video materials and a third cohort (*N = 33*) were not sent any materials. On arrival participants completed a simple knowledge test, a questionnaire estimating the time they spent preparing for the placement and a self-reported knowledge score. The teaching on placement was the same for all cohorts. At the conclusion of each placement participants completed a challenging knowledge test, a clinical skills test and repeated self-reported knowledge scores.

**Results:**

Eighty seven percent of students receiving specified materials claimed to complete pre-placement work compared to 70% of those receiving learning outcomes alone (*p* = 0.05). Students receiving learning materials scored higher in the post-placement tests of knowledge (*p* < 0.001), 74.8% (72.4–77.2%) vs 63.6% (95%CI 59.3–67.9%) and skills (*p* = 0.04), 86.9% (83.9–89.9%) vs 81.3% (77.2–85.4%). Students using video resources outperformed students using written materials in their visual acuity assessment test (*p* = 0.03), 90.4% (86.6–94.2%) vs 83.6% (80.1–87.1%) whilst those receiving written rather than video material performed better in the end of placement knowledge test (*p* = 0.03), 77.7% (74.3–81.1%) vs 72.0% (68.9–75.1%).

**Conclusion:**

This study showed that providing pre-placement learning materials improves undergraduates’ commitment and achievement. Written materials better facilitate knowledge acquisition while video materials preferentially promote skill acquisition. This is a novel demonstration within ophthalmology and can help address the imbalance between the expectations placed on undergraduates and the resources committed to ensuring they are met.

## Background

The modernisation of undergraduate medical curricula has frequently involved a reduction in the course time dedicated to ophthalmology across the globe [1–4]. This is thought to stem from curriculum designers striving to cover a growing curriculum and to grant their graduates access to the increasingly disparate careers of primary care generalist, specialist and researcher [5]. Interventions such as the establishment of a standardised undergraduate curriculum and a minimum clinical exposure time from the International Council of Ophthalmology have not reversed this long-reported decline [6, 7]. There are consequences for patient care in this trend as primary care physicians consistently report low levels of confidence in managing eye conditions with a majority feeling their undergraduate ophthalmology education was inadequate [8–10]. If increasing undergraduate ophthalmology exposure is untenable, then a shift in focus towards optimising the efficacy of the limited time learners have is likely to be more productive.

The nature and content of a clinical placement is of central importance to learners’ educational outcomes, but is dependent on the host institution’s ability to designate appropriate resources. The resources dedicated to undergraduate ophthalmology education are often minimal and a recent systematic review has called for research to focus on improving the efficiency of teaching rather than demanding a greater share of teaching time [11]. The theoretical basis of this goal is to minimise the extrinsic and germane cognitive load that learners experience during their placement in order to maximise the progress they can make in the time available [12]. To achieve this, students need to be made as receptive as possible to the learning outcomes prior to starting a placement. A key contributor to this is the pre-training principle; where learners gain more knowledge from a given learning experience if they have prior exposure to its content and how to process it [13]. In their meta-analysis of 128 studies into the impact of pre-training for training systems ranging from typing to police operations, Mesmer-Magnus et al. demonstrated a significant impact for pre-training on cognitive, skill and affective learning outcomes [14].

To test the relevance of pre-training theory to undergraduate ophthalmology we present a pragmatic observational study of final year medical students. We hypothesise that medical undergraduates demonstrate greater learning over the course of a short ophthalmology placement if directed towards relevant learning materials beforehand. Our primary outcome measure is the end of placement test score as it is most representative of the tools used to assess undergraduate ophthalmology competency by medical schools. Secondary outcomes include pre-placement work completion and duration, initial test scores, in-placement test scores, clinical supervisor impression, self-reported scores and end of placement examination skills test scores. We will also explore the relative impacts of directing learners toward video and textual resources as prior work in undergraduate ophthalmology has suggested a greater efficiency of learning from video materials [15].

## Methods

Ninety-eight final year medical students attended an ophthalmology department at a tertiary centre for a three-day clinical placement between January and May of 2017. Each three-day placement was attended by groups of six or seven students and their placement was an equal mix of classroom and workplace teaching, alternating between the two environments each day over two sessions on the first day and four sessions on the second and third day. Classroom sessions were broken down into short lectures and practical role play history and examination exercises. Workplace sessions were delivered by members of the clinical team through one to one shadowing in the emergency eye department, general ophthalmology clinics and subspecialty ophthalmology clinics. All placements were managed by a single clinical teaching fellow and involved the same clinics to ensure the content of each placement remained constant throughout the study period. As in this case, clinical teaching fellows are typically junior doctors with a mix of clinical and education duties. Typically, they have completed at least 2 years of postgraduate practice and may or may not have committed to a certain specialty. One week prior to attending each student was emailed a welcome pack composed of an introductory letter, a timetable for the placement, a list of core conditions and a request that students undertake 90 min of work in preparation for a test upon arrival. The students were also notified of the education research project and asked if they were willing for their anonymised data to be used, this written request was repeated verbally at the outset of each placement. We developed the welcome pack on two occasions over the five-month period, but the 1 week advance with which students received it remained constant throughout. Firstly we attached written materials which covered core ophthalmology content and subsequently we replaced these written materials with links to educational videos covering the same content. These written and video materials are publicly available and similar welcome packs could easily be replicated at other institutions [16]. No other interventions were made.

To remove bias from diffusion of treatment we collected data from consecutive groups who received the welcome pack in one of the three stages of development; no materials, written materials and video materials. At the outset of the placement each student completed a 45 mark test (one mark per question) covering the basics of ocular anatomy, retinopathy, visual pathway, red eye and visual loss. Students also completed hard copies of five-point Likert scale questionnaires with only numerical extremes marked by text (5 positive, 1 negative) marked. These indicated their perception of their own ophthalmology knowledge and their interest to pursue it as a career. During the placement ten-point Likert scales were emailed to consultants who had provided individual teaching in clinics to gain their impression of student knowledge and engagement, again only numerical extremes were translated to text (10 positive, 1 negative). These consultant supervisors were blinded to pre-material allocation. As an objective measure of performance during the placement, groups were also tested on the content of seminars at their conclusion using Turning Point™ (Turning Technologies™, Ohio, USA). These took the form of 24 five-choice multiple choice questions delivered over four classroom sessions (one mark per question). At the conclusion of the placement each student completed a more challenging exam with 22 four-choice multiple choice questions covering the core conditions stated in their welcome pack (one mark per question). They were also asked to rate their own ophthalmology knowledge and career interest again using the same Likert scale from the initial assessment. Each student was then tested on their ability to assess visual acuity with a simulated patient using an objective structured clinical examination (OSCE). At the conclusion of the final placement all 98 students were invited to complete a hard copy of a ten-point Likert questionnaire on their perceived efficacy of different learning strategies for the acquisition of knowledge and skill. As before, only numerical extremes were translated to text. At this point all students had been made aware of the written and video learning materials used over the 5 months through their online learning management system.

A prospective power calculation was not performed as the study was built around the host ophthalmology teaching programme and so cohort size was limited to a single final year group. Data were analysed using SPSS v.24 (IBM Corporation, New York, USA) comparing the performance of students given specified pre-materials with those who were not. An intention-to-treat approach was used for comparative tests as the study aimed to produce pragmatic data reflecting the impact of the intervention. In some settings additional insights were gained from as-treated analysis, these comparisons are specified. The impact of written and video pre-materials was also compared. When comparing two groups, two-tailed student t-tests or Mann-Whitney u-tests were used for parametric and non-parametric data respectively. As the primary hypothesis this study aimed to test was whether or not pre-placement materials of any kind were beneficial, we initially compared students receiving no materials to those receiving video or written materials. To address our secondary hypothesis that the efficacy of video and written materials differ we then went on to perform a direct comparison between written and video materials for variables that had proved significant in the first analysis. To test correlations, Pearson r and Spearman’s rho were used for parametric and non-parametric data respectively.

## Results

### Pre-placement learning materials

**Fig. 1:**
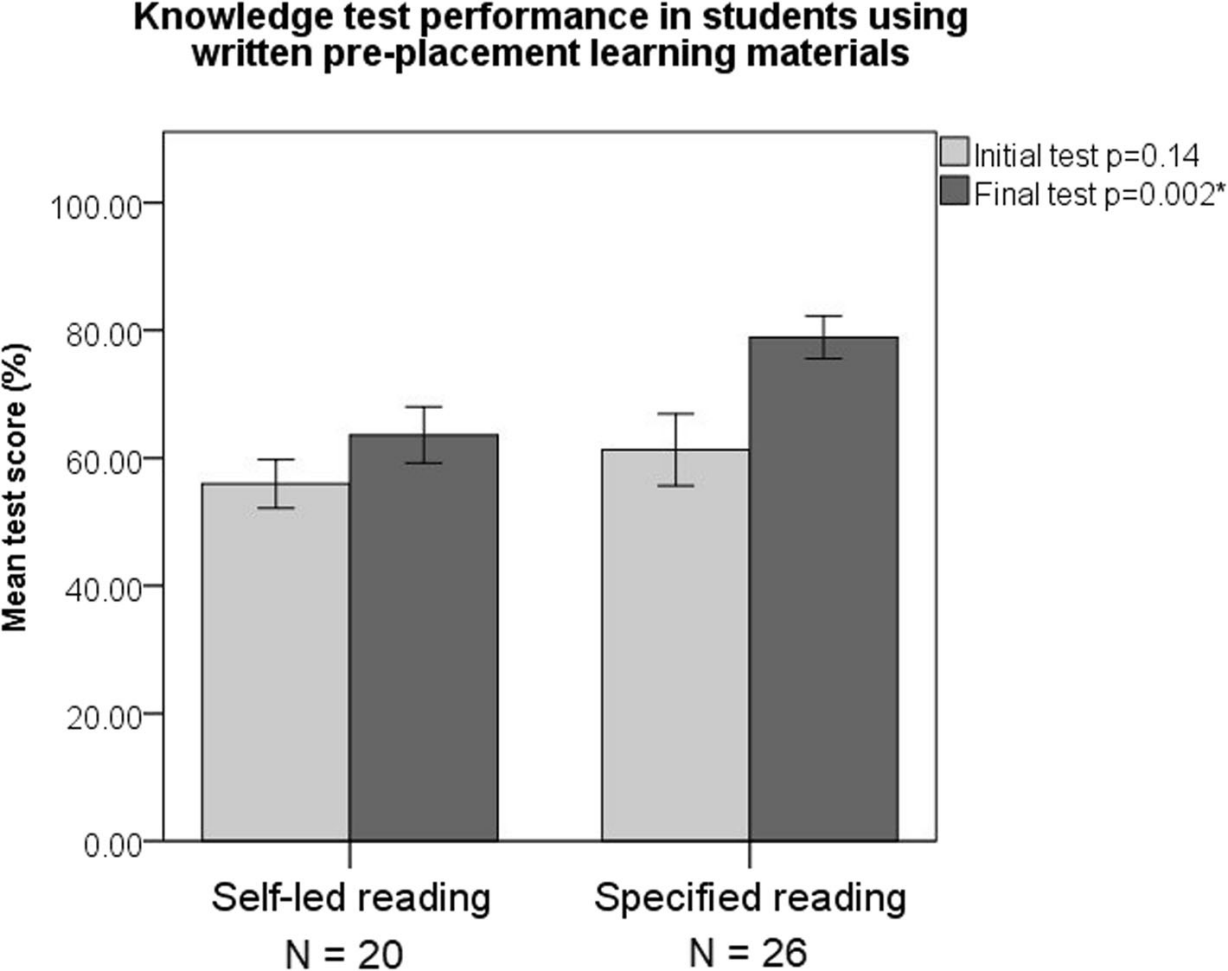
Chart displaying an as-treated analysis of student performance on two different ophthalmology knowledge tests at the outset of the placement and at its conclusion. Chart shows means with 95% confidence intervals, *p* values calculated with Mann-Whitney u-tests

Ninety five percent of students (*N* = 93) volunteered whether or not they had done pre-placement work (Table 1). Students who had been given specified written or video materials were more likely (*p* = 0.05) to prepare for the placement (87%) than those who had not (70%). Whether these resources were video or written had no significant impact on the likelihood that they would be used (*p* = 0.11). Of the 33 students not sent specified resources 61% (*N* = 20) identified and read their own written materials and 9% (*N = 3*) identified and viewed video materials. In an as-treated analysis students identifying their own written resources performed worse in both initial and final knowledge tests compared to students receiving specified written resources (Fig. 1).

**Table 1:** Table showing student resource allocation and distribution of student-reported pre-placement work completed

	No specified materials *N* = 33	Written materials received *N* = 33	Video materials received *N* = 32	Total *N* = 98
Declined comment	0 (0%)	2 (6%)	3 (9%)	5 (5%)
No work done	10 (30%)	2 (6%)	6 (19%)	18 (18%)
Written materials used	20 (61%)	26 (79%)	1 (3%)	47 (48%)
Video materials used	1 (3%)	0 (0%)	19 (59%)	20 (20%)
Audio materials used	1 (3%)	2 (6%)	0 (0%)	3 (3%)
Both video and written materials used	1 (3%)	1 (3%)	3 (9%)	5 (5%)

Of the students claiming completion of pre-placement work the time spent was not significantly affected by having had resources specified to them (Table 2). For this comparison students denying completion of pre-placement work were assigned a time of zero minutes. The difference in score on the initial knowledge test was also not significantly different between the two groups (*p* = 0.15). However, a five point agreement Likert scale with the statement ‘my knowledge of ophthalmology is adequate to pass my finals examination’ showed students’ perception of their own knowledge was improved by pre-placement materials (*p* = 0.005). Final knowledge and skills test scores, final self-reported knowledge ratings and supervisor reported knowledge were all found to be significantly higher in students who had received specified pre-placement learning materials (Table 2). However, no significant differences were found in knowledge retention immediately after seminars. Supervisor reported student engagement levels were also found to be equivalent between groups (*p* = 0.78).

**Table 2:** Table showing differences in performance between students who received specified pre-placement learning materials and those who did not

	No PPR	95% *CI*	Specified PPR	95% *CI*	*p* value
Mean time on pre-placement work (mins)	51	43.3–58.7	61.9	39.0–84.8	0.15
Mean initial knowledge test score (%)	56	52.3–59.7	60.4	56.8–64.0	0.12
Mean initial self-reported knowledge rating/5	1.7	1.4–2.0	2.3	2.1–2.5	0.005*
Mean supervisor knowledge rating/10	6.4	5.7–7.1	7.5	7.0–8.0	0.006*
Mean supervisor engagement rating/10	8.6	8.2–9	8.5	8.1–8.9	0.78
Mean post-seminar interactive test score (%)	70.4	61.6–79.2	74.5	70.5–78.5	0.89
Mean final knowledge test score (%)	63.6	59.3–67.9	74.8	72.4–77.2	< 0.001*
Mean final self-reported knowledge rating/5	3.8	3.5–4.1	4.3	4.1–4.5	0.001*
Mean OSCE score (%)	81.3	77.2–85.4	86.9	83.9–89.9	0.04*

*p* values calculated using two-tailed t-tests for parametric data and Mann-Whitney u-tests for non-parametric data. *OSCE* Objective structured clinical examination. *CI* Confidence interval. *PPR* Pre-placement resources. * = statistical significance defined as *p* < 0.05

### Video versus written pre-placement learning materials

Of the 33 students receiving written pre-placement learning materials 82% (*N* = 27) used them whereas 69% (*N* = 22) of the 32 students receiving video materials made use of them. Of the five parameters found to be significantly impacted by specifying learning resources of some kind, only the end of placement knowledge test score was significant when comparing video and written pre-materials(Table 3). Students receiving written materials scored a mean of 77.7% compared to 72.0% among students receiving video materials (*p* = 0.03). With an as-treated analysis, students that went on to use video materials had a mean OSCE score of 90.4% (95% CI: 86.4–90.4%) compared to 83.6% (95% *CI*: 80.1–87.1%) for students who went on to use written materials (*p* = 0.03).

**Table 3:** Comparison of the impact of specifying video and written pre-placement learning materials in parameters where specifying written or video learning materials was found to have a significant impact

	Video LRS	95% *CI*	Written LRS	95% *CI*	*p* value
Mean initial self-reported knowledge rating/5	2.4	2.1–2.7	2.1	1.8–2.4	0.14
Mean supervisor knowledge rating/10	7.5	6.9–8.1	7.6	6.9–8.3	0.70
Mean final knowledge test score (%)	72.0	68.9–75.1	77.7	74.3–81.1	0.03*
Mean final self-reported knowledge rating/5	4.2	3.9–4.5	4.4	4.2–4.6	0.47
Mean OSCE score (%)	88.1	84.0–92.2	86.0	81.6–90.4	0.53

*p* values calculated using two-tailed t-tests for parametric data and Mann-Whitney u-tests for non-parametric data. *OSCE* Objective structured clinical examination. *CI* Confidence interval. *LRS* Learning resources specified. * = statistical significance defined as *p* < 0.05

This suggestion of video materials’ superiority for skill acquisition was supported by student perception of the relative value of these modalities. Fifty-three students (54%) completed 10-point Likert questionnaires demonstrating their agreement with four statements; ‘video/written learning materials are most effective for knowledge/skill acquisition’. Using a paired sign test, on account of the non-parametric and skewed nature of the data, there was no significant difference in student perception of video and written materials for knowledge acquisition. However, video materials were considered significantly better for skill acquisition (Table 4).

**Table 4:** Student reported perception of video and written learning material efficacy for knowledge and skill acquisition

	Knowledge			Skill		
	Mean	95% *CI*	*p* value	Mean	95% *CI*	*p* value
Written materials	5.4	4.9–5.9	0.12	4.8	4.3–5.4	< 0.001
Video materials	5.8	5.3–6.3		6.6	6.2–7.1	

Data are taken from ten-point Likert scales with a higher score representing greater perceived efficacy. *p* values are calculated with paired sign tests

### Student performance prediction

**Fig. 2:**
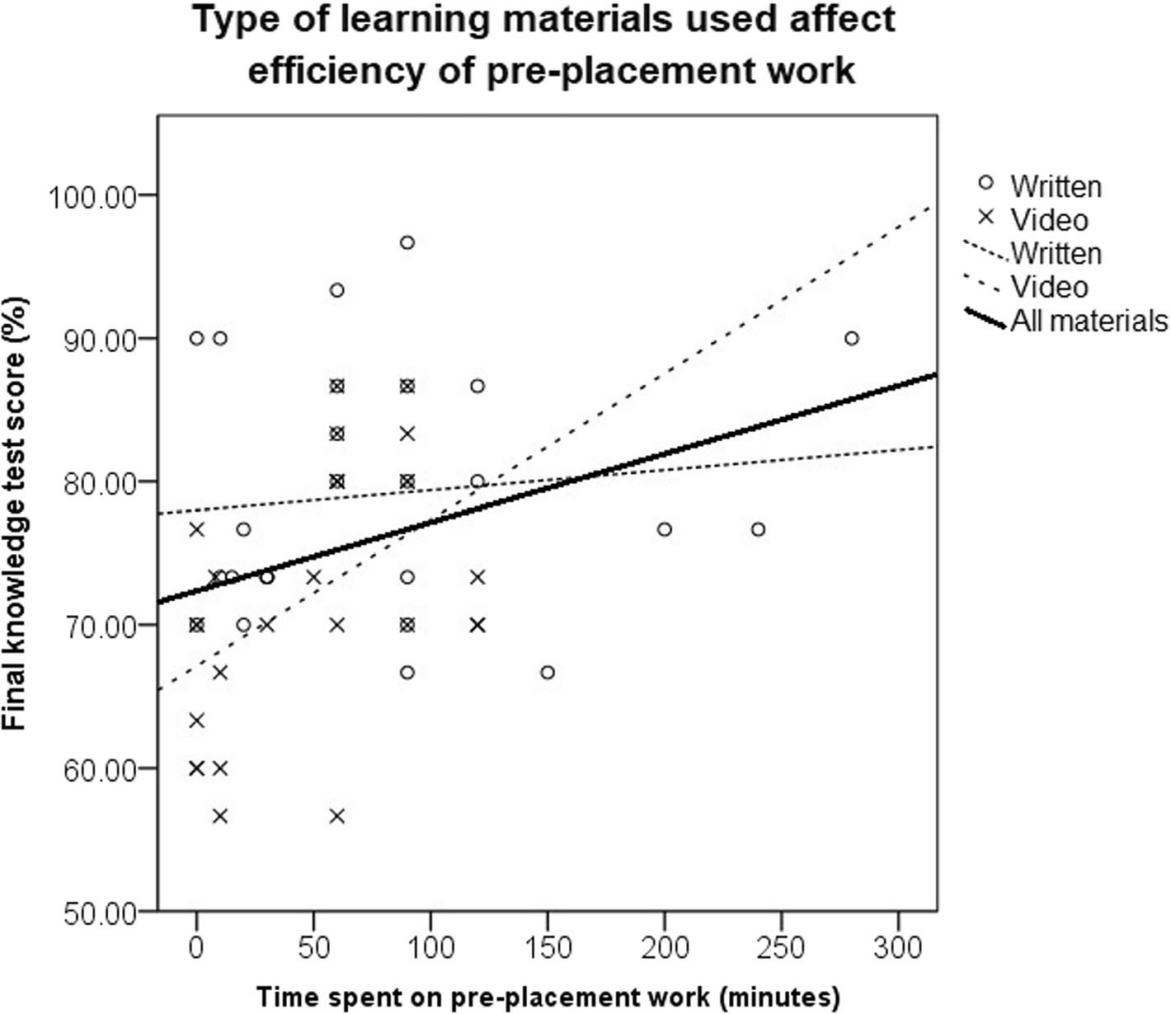
Scatter plot of the time students claimed to have spent on pre-placement work and their final knowledge test score with trend lines. Students using video resources are represented by crosses whilst those using written resources are represented by circles

There was a significant positive correlation (r = 0.316) between the time students claimed they had worked prior to their placement and their performance at the end of placement knowledge test (*p* = 0.003). When applying an as-treated analysis to these data separately for video and written resources, the correlation coefficients were 0.455 (*p* = 0.06) and 0.239 (*p* = 0.13) respectively (Fig. 2). There was also a positive but non-significant relationship between supervisor impression of student knowledge and performance in the end of placement knowledge test (*p* = 0.13).

## Discussion

This pragmatic single centre study shows that the provision of learning materials prior to undergraduate ophthalmology clinical placements significantly improved subjective and objective measures of knowledge and skill performance. This effect is likely due to both the demonstrated increased engagement with pre-training by students when materials were specified and also the higher efficacy of teacher selected materials relative to student selected materials. As few students in our study independently sourced video materials we were unable to evaluate the efficacy of student sourced video materials. However, evidence from ophthalmology and other areas of healthcare suggest that clinical skills videos selected to meet student needs are also more effective than video materials that students source independently [17, 18]. Our data suggest that written materials yield better results on knowledge tests than video materials if student time is unlimited. However, if students are only willing or able to commit a short period of time to self-directed learning it seems that video materials may have a greater yield (Fig. 2). This is consistent with a randomised controlled trial (RCT) in ophthalmology undergraduate education from Steedman et al. which demonstrated equivalent test results following a shorter work duration from students using multimedia learning compared to textual materials alone [15]. Whilst intention-to-treat analysis did not find a significant difference in OSCE performance between recipients of video and written materials, a comparison based on what materials individuals actually went on to use did suggest an objective advantage in clinic skill learning for video materials. This is also supported by subjective data that video materials are more effective than written materials in helping learners acquire skills (Table 4). These findings are consistent with work in endocrinology showing improvement in undergraduate clinical skill performance with the availability of specified video materials [19].

The proof of our hypothesis that pre-placement materials improve learning efficiency on an undergraduate ophthalmology placement is informative but not unexpected. The real value of the outcome is the reproducibility of the means by which it was achieved. To minimise barriers to teachers implementing similar approaches both the welcome pack and learning resources used are publicly available (Additional files 1, 2 and 3) [16]. In considering feasibility of the distribution of pre-placement materials at any given institution it is also important to address the barriers to success from the learner perspective. Reid et al. recently performed thematic analysis on interviews with Irish medical undergraduates regarding a year of self-led e-learning [20]. The three major themes of barriers to engagement were a sense of being cheated out of higher quality traditional teaching methods, the ease with which attention could fail to be paid to audio and video materials and a sense of being overwhelmed at a large bank of seemingly unstructured materials [20]. We attempted to address these barriers by making our learning materials an adjuvant rather than an alternative to traditional teaching and by requesting a short period of commitment from learners. The mean of 62 min (median = 55, interquartile range 10–90) that students who received pre-placement materials claimed to have spent compared to the 90 min requested of them suggest these attempts were at least in part successful. Student feedback placed great value on the welcome pack that had been sent 1 week prior to attending the placement, as it gave them an early sense that their learning had been carefully considered. This helped to persuade them into committing their own time. It may be that a more refined pre-placement welcome pack could win even greater time commitments from student and elicit further learning benefits. However, student engagement may not be so readily won if pre-placement materials become ubiquitous for all medical student placements. If this proves to be the case it may be best to prioritise areas of the curriculum where student exposure is limited.

In considering the application of these data it is important to appreciate that the specialty of ophthalmology is just one of many areas in the undergraduate medical curriculum where student exposure is limited. Plastic surgery, neurosurgery, otolaryngology and cardiothoracic surgery are all examples of specialties with little presence in timetabled teaching where students could benefit from an increase of teaching efficiency if not quantity [21–24]. Another transferrable theme that was frequently voiced by students in open space feedback through this study was the perceived benefit of the provision of a clinical member of staff dedicated to their teaching. These student comments are consistent with UK student surveys on clinical teaching fellows who are perceived to deliver higher quality teaching, be more punctual and have a clearer understanding of students’ learning needs compared to full time clinicians [25, 26]. In our experience the service provision capacity of the department was also improved by the introduction of a teaching fellow as more experienced clinical staff, who have previously shared teaching responsibilities, were no longer required to be withdrawn from clinical duties. If the teaching demands on a department are great enough, as in this case, the consequent increase in clinical capacity can offset the cost of employing a full or part time teaching fellow. Disruption to service provision can be reduced while teaching quality is improved at little or no cost.

The study was limited by its design as a longitudinal quality improvement project. This was chosen over a RCT as it would not have been feasible to stop students sharing learning materials with peers in different groups; a recognised limitation of RCT in medical education research [27]. Our approach also meant that the whole year group was able to access learning resources as our links to learning resources were developed, albeit at varying time points relative to their placement. The study design also prohibited any control over the cohort size as it was built around an established annual teaching programme. Any attempt to extend beyond a year would have raised issues of bias as the teaching fellow delivering the course changes annually and would also introduce ethical issues, as learning materials with proven efficacy would be withheld from students in the second year. This limits the external validity of the study as only statistically significant differences are reported rather than prospectively defined ‘clinical’ significance.

The reader should also consider the discrepancy between what learning materials were sent to students and which materials they went on to use. We chose to analyse the variable within the control of the education staff, namely the materials sent to the students, in order to maximise the external validity of our data. It is also true that the self-reported outcomes, such as material type used, time spent working and perceived knowledge levels cannot be assumed to be accurate. This limitation is likely to be greatest for the claims students made of how much time they worked, as this is likely to be influenced by other factors such as student confidence and the degree to which they did not want to disappoint teaching staff. Whilst this may limit the accuracy of the measure, these confounding factors ought to have been distributed randomly between the three groups so should not bias the outcome. An objective means of measuring the quantity and quality of pre-placement work each student performed would be technically and ethically challenging and would impose bias from the Hawthorn effect. For self and supervisor reported knowledge outcomes, it is true that they do not hold the same objectivity as test scores but student confidence and esteem is also an important outcome of education and these outcomes complement rather than duplicate the test scores reported. When considering the analysis some of the significance demonstrated is not maintained following Bonferroni correction. The risk of type 1 error should be borne in mind but amongst many others our primary outcome of final test score remains significantly dependent on pre-placement material provision following Bonferroni correction.

## Conclusion

This pragmatic comparative study found that the provision of specified learning materials prior to a short ophthalmology clinical placement augmented student performance at its conclusion. Written learning materials improved knowledge test performance to a greater extent than video materials, whilst video materials were superior for clinical skill development. Judicious selection of an appropriate learning material format for given learning outcomes can optimise the educational efficiency of teacher time expenditure.

## Supplementary information

**Additional file 1.** Welcome pack sent to the first cohort of students who did not receive any prescribed pre-placement materials.

**Additional file 2.** Welcome pack sent to the second cohort of students who received links to specified written materials.

**Additional file 3.** Welcome pack sent to the third cohort of students who received links to specified video materials

## Data Availability

The datasets generated and/or analysed during the current study are not publicly available as data sharing was not consented for or included in the protocol reviewed by local ethics boards.

## Abbreviations

CI: Confidence Interval
OSCE: Objective Structured Clinical Examination
RCT: Randomised Controlled Trial
