# Supplementary material for: A non-randomised trial of video and written educational adjuncts in undergraduate ophthalmology
Source: BMC Med Educ. 2020 Jan 9;20:10. doi: 10.1186/s12909-019-1923-1 (PMC6953281; doi:10.1186/s12909-019-1923-1)
Supplement: Supplementary file 1 — Additional file 1. Welcome pack sent to the first cohort of students who did not receive any prescribed pre-placement materials. [file 12909_2019_1923_MOESM1_ESM.docx]

HBP Ophthalmology 2017

Newcastle Eye Centre

Claremont Wing

Royal Victoria Infirmary

Newcastle upon Tyne Hospitals’ NHS Foundation Trust

Queen Victoria Road

Newcastle upon Tyne

NE1 4LP

# Welcome to Newcastle Eye Centre.

Ophthalmology remains one of those daunting topics which can never be covered in full in three days – but with a firm grasp of the fundamentals, we hope that we can show you that eyes are not scary and that you will be able to safely manage any eye condition which you come across in your career.

Necessarily, the learning objectives list is lengthy but this just reflects the breadth of the specialty. We are fortunate to be both physicians and surgeons and we see the newborn to the advanced in years.

Gratifyingly, the majority of the interventions in Ophthalmology are sight preserving and in many cases, have huge positive impact on our patient’s lives.

NEC in particular is at the forefront of ophthalmic service provision and research. We have an established corneal stem cell transplantation programme and we are the regional orbital surgery and anophthalmic socket service provider. We are one of the largest treatment centres for age-related macular degeneration in the United Kingdom as well as one of the busiest cataract treatment centres. We are the ophthalmic opinion for the Great North Children’s Hospital and are the only unit in the region with Medical Ophthalmologists. There are regular MDTs with Dermatologists, Endocrinologists, Neurosurgeons, Plastic Surgeons, Maxillo-facial Surgeons and we are fully supported by Optometrists, Orthoptists and Specialist Nurses. The Ophthalmology department has its own dedicated Eye Emergency Department which sees approximately 25000 patients per year and we have 5 dedicated Eye Theatres here in the Claremont Wing. It’s fair to say that we are quite busy!

We are always asked to set a station at the MOSLERS and more likely than not, it will be something that would have been covered in the three days with us. We don’t expect you to become experts in the short time that you are with us, but if you walk away with a better idea of what to do when a patient comes to you with an eye problem, then it would have been time well spent.

With all good wishes

Gordon Lau

Consultant Ophthalmologist

Undergraduate Teaching Lead (Ophthalmology)

HBP Ophthalmology 2017

# Learning aims:

**To gain fundamental knowledge to safely deal with ophthalmic disease presentations and be able to report findings when referring patients for specialist opinion.**

**To develop appreciation of the ophthalmic manifestations of systemic disease.**

**To give an insight into Ophthalmology and encourage pursuit of the specialty as a career.**

# Learning outcomes:

## Learners will identify the components of basic ocular and visual pathway anatomy and be able to describe their physiological function.

Label the following structures on a diagram; cornea, conjunctiva, anterior chamber, iris, pupil, iridocorneal angle, lens, suspensory ligaments, vitreous humour, retina, optic disc, optic nerve, and sclera

Summarise the role of the cornea, conjunctiva, iris, pupil, iridocorneal angle, lens, retina, optic disc and optic nerve

Summarise what uveal tissue is and explain the anatomical categorisation of uveitis

Identify the 6 extraocular muscles and explain the way each moves the eye and how they are innervated

Recall the 5 main branches of the facial nerve and their distribution

Identify the site of a lesion in the visual pathway in the following visual defects – monocular total field loss, bitemporal homonymous hemianopia, homonymous hemianopia/quadrantinopia and altitudinal field loss

## Discuss the presentation, investigation and treatment of core conditions

Periorbital and orbital cellulitis

Ptosis

Eyelid lesions

Blepharitis

Facial nerve palsy; lower vs upper motor neurone lesions

Third nerve palsy

Horner’s Syndrome

Herpes Zoster Ophthalmicus

Cornea and conjunctiva

Dry eye; involutional, Sjogren’s and blepharitis

Corneal abrasion

Corneal ulcer/infective keratitis

Chemical injury – acid or alkali

Corneal foreign body e.g. metal

Penetrating corneal injury

Conjunctivitis; bacterial, allergic and viral

Subconjunctival haemorrhage

Anterior uveitis

Traumatic mydriasis/penetrating trauma

Acute angle closure glaucoma

Open angle glaucoma

Presbyopia

Cataract

Leukocoria in infants

Vitreous haemorrhage

Endophthalmitis

Posterior vitreous detachment

Retinal tear/detachment

Retinal vascular occlusion

Diabetic retinopathy

Age related macular degeneration

Giant cell arteritis

Optic disc swelling; unilateral and bilateral

Strabismus; separate from cranial nerve palsy

## Recall the ocular manifestations of core systemic diseases

Diabetes

Hypertension

Rheumatoid arthritis

Wegener’s granulomatosis

HIV

Thyroid disease

Ankylosing spondylitis

Multiple sclerosis

## Construct a safe immediate management plan for ophthalmic emergencies and apply a systematic algorithm to decide whether specialist referral is required

Acute angle closure glaucoma

Chemical injury

Endophthalmitis

Corneal ulcer

Giant cell arteritis

Penetrating trauma

Third nerve palsy

Sixth nerve palsy

Horner’s Syndrome

Retinal detachment

## Take a concise ophthalmic history and apply it to generate a differential diagnosis

Blurred vision

Bits of vision missing

Double vision

Foreign body sensation

Pain

Flashes of light

Floaters

Photophobia

Discharge, watery or mucus

Distortion

Loss of colour

Glare

## Competently perform fundamental ophthalmic examination techniques

Accurately assess visual acuity and document, including use of pinhole

Discuss strategies to assess vision when no Snellen chart available or vision deemed unassessable e.g. illiteracy, infants, malingering

Determine the absence or presence of a relative afferent pupillary defect

Perform a cover test and interpret the findings

Accurately assess visual fields by confrontation and be familiar static and kinetic perimetry

Examine eye movements and document clearly

Eversion of eyelids

Use the slit-lamp biomicroscope to assess the ocular surface

Use topical fluorescein, anaesthetic, mydriatics and antibiotics appropriately and discourage the inappropriate use of topical anaesthetic and topical steroid

Use the direct ophthalmoscope to visualise the optic disc and retina being able to comment on the presence or absence of optic disc swelling, massive retinal detachment, diabetic retinopathy or hypertensive retinopathy

## Describe the impact of visual impairment

Driving and occupation

Mental health; depression, Charles-Bonnet Syndrome

Loss of independence at any age

HBP Ophthalmology 2017

# Compulsory pre-placement study

Familiarity with this material will be tested at the beginning of this placement. Though we are aware you have a range of study demands to balance we simply cannot offer you the opportunity to meet the above learning outcomes without your help. For the majority of you this placement will represent the last specialist led ophthalmic tuition you will receive in your entire career and we want to prepare you not just for ophthalmic MOSLER cases in finals but for the decades of practice that will follow.

We would recommend a minimum of 90 minutes work prior to starting your placement covering basic content around:

- Eye anatomy
- Retinal pathology
- Eye infections
- Ophthalmic examination techniques

HBP Ophthalmology 2017

# Assessment

To ensure you get what you need from your time with us and to provide some structure we have set a number of cases we would like you **either** to take a history from, examine or discuss with an ophthalmologist (tick the appropriate boxes below). We would encourage you to fill the table in as much as you can but as a minimum require you to have touched on each of the below conditions and to gain a signature from the supervising doctor or nurse practitioner.

| **Condition** | **History** | **Examination** | **Discussion** | **Tutor’s signature** |
| --- | --- | --- | --- | --- |
| Anterior Uveitis |  |  |  |  |
| Blepharitis |  |  |  |  |
| Cataract |  |  |  |  |
| Conjunctivitis |  |  |  |  |
| Foreign Body |  |  |  |  |
| Glaucoma |  |  |  |  |
| Keratitis |  |  |  |  |
| Posterior Vitreous Detachment |  |  |  |  |

Along with this log a short test will be sat at the end of the week to ensure the placement learning outcomes have been met.
